# Homeotic Genes: Clustering, Modularity, and Diversity

**DOI:** 10.3389/fcell.2021.718308

**Published:** 2021-08-11

**Authors:** Nikhil Hajirnis, Rakesh K. Mishra

**Affiliations:** ^1^CSIR – Centre for Cellular and Molecular Biology (CCMB), Hyderabad, India; ^2^AcSIR – Academy of Scientific and Innovative Research, Ghaziabad, India; ^3^Tata Institute for Genetics and Society (TIGS), Bangalore, India

**Keywords:** hox, evolution, patterning, gene regulation, bithorax complex, vertebrate hox, modularity and adaptability, homeotic transformation

## Abstract

Hox genes code for transcription factors and are evolutionarily conserved. They regulate a plethora of downstream targets to define the anterior-posterior (AP) body axis of a developing bilaterian embryo. Early work suggested a possible role of clustering and ordering of *Hox* to regulate their expression in a spatially restricted manner along the AP axis. However, the recent availability of many genome assemblies for different organisms uncovered several examples that defy this constraint. With recent advancements in genomics, the current review discusses the arrangement of *Hox* in various organisms. Further, we revisit their discovery and regulation in *Drosophila melanogaster*. We also review their regulation in different arthropods and vertebrates, with a significant focus on *Hox* expression in the crustacean *Parahyale hawaiensis*. It is noteworthy that subtle changes in the levels of Hox gene expression can contribute to the development of novel features in an organism. We, therefore, delve into the distinct regulation of these genes during primary axis formation, segment identity, and extra-embryonic roles such as in the formation of hair follicles or misregulation leading to cancer. Toward the end of each section, we emphasize the possibilities of several experiments involving various organisms, owing to the advancements in the field of genomics and CRISPR-based genome engineering. Overall, we present a holistic view of the functioning of *Hox* in the animal world.

## Introduction

The development of an animal progresses three-dimensionally across anterior-posterior (AP), dorsal-ventral (DV), and left-right (LR) axes. A combination of various transcription factors, epigenetic regulators, cell receptors, and signaling molecules are involved in the overall development of an organism (François et al., 1994; Beddington and Robertson, 1999; Levin, 2005; Peel et al., 2005; Dequéant and Pourquié, 2008; Basson, 2012; Perrimon et al., 2012; Coutelis et al., 2013; Berenguer et al., 2020). Homeotic genes or *Hox* are one of the significant contributors to bilaterian development and are evolutionarily conserved. They are often present in clusters and code for transcription factors (HOX) that act on the downstream genes to provide identity to developing segments along the AP axis of a bilaterian embryo (Akam et al., 1988; Akam, 1998; Lewis, 1998).

A series of genetic crossings and recombinations lead to the discovery of *Hox* in *Drosophila melanogaster*. Interestingly, all the genes were mapped to the right arm of the third chromosome in the fruit fly. Further, the genes were clustered together in two complexes of ∼300 Kb each – Antennapedia complex (ANT-C) and bithorax complex (BX-C), named after the initial phenotypes obtained for different alleles in both complexes. Strikingly, the arrangement of these genes on the chromosome followed an order that was collinear to the segments affected in mutants from anterior to posterior direction. Thus, in the fly, the concept of spatial collinearity was established for *Hox* expression (Lewis, 1978, 1998; Kaufman et al., 1990). This expression is under the control of several *cis*-regulatory elements (CREs) that cluster together to form *cis*-regulatory modules (CRMs) (Peifer and Bender, 1986; Peifer et al., 1987; Celniker et al., 1989; Martin et al., 1995; Maeda, 2009; Chopra, 2011; Bekiaris et al., 2018). Series of discoveries toward the turn of the 20th century showed the presence of *Hox* in all bilaterians and even in cnidarians (Ferrier et al., 2000; Kourakis and Martindale, 2001; Ferrier and Minguillón, 2003; Ikuta et al., 2004; Duboule, 2007; Mooi and David, 2008; Mallo et al., 2010; Ikuta, 2011; Janssen et al., 2014; Fritsch et al., 2015; Schiemann et al., 2017; Wanninger and Wollesen, 2019; Nong et al., 2020). The transcription factors coded by these genes have a conserved helix-turn-helix motif-containing DNA binding domain. The domain binds to DNA in a sequence-specific manner and is called the homeodomain due to its discovery in the factors coded by *Hox*. Many transcription factors in addition to HOX across animals, plants, and fungi have the homeodomain (McGinnis et al., 1984b, a; Scott and Weiner, 1984; Suzuki and Yagi, 1994; Williams, 1998; Holland, 2001; Holland et al., 2007; Son et al., 2020). Therefore, all Hox genes are homeobox genes, but all homeobox genes are not Hox genes. The complex interplay of HOX proteins with other players in the system contributes toward diversity in the animal kingdom (Akam et al., 1988; Akam, 1998; Lewis, 2007; Holland, 2015; Rogers, 2020).

In the current review, we discuss the arrangement and copies of Hox genes in different organisms. We then revisit their discovery and regulation in *D. melanogaster*, subsequently commenting upon their *cis*-regulation in vertebrates. Further, the review highlights the presence of these genes in other arthropods and their expansion in vertebrates, with a significant focus on *Hox* expression in *Parhyale hawaiensis*. The crustacean is an emerging model organism with established gene-editing techniques such as CRISPR-Cas9 to decipher the role of *Hox*, adding them to the league of other classical models, including fruit fly, zebrafish, or mouse (Martin et al., 2016; Sun and Patel, 2019). Notably, the function of these genes is not limited to segment identity and homeotic transformations (Castelli-Gair Hombría and Lovegrove, 2003). We also underline many upcoming reports that describe their role in tissue homeostasis, embryonic cell fate determination, organogenesis including abdominal epithelium in flies or hair follicles in mammals, maintenance of stem cells niche, and misregulation leading to cancer (Lewis, 2000; Awgulewitsch, 2003; Shah and Sukumar, 2010; Estacio-Gómez and Díaz-Benjumea, 2014; Singh and Mishra, 2014; Domsch et al., 2019). Toward the end of each section, we emphasize the possibilities of novel experiments to understand the regulation and functioning of Hox genes in different organisms. This largely owes to the recent advances in genomics and genome editing technologies, including CRISPR-Cas9. We thus present a bird’ eye view of the Hox field and prospective investigations required to understand their role in various organisms.

## *Hox* Clusters: Arrangement, Positioning, and Duplications

The animal kingdom has diverse body forms, symmetries, and developmental axes. *Hox* are one of the key contributors to this diversity as they provide identity to different segments during embryonic development, are involved in tissue homeostasis and organ positioning, and help in maintaining cellular identities post-embryonic development (Lewis, 2000; Castelli-Gair Hombría and Lovegrove, 2003; Lovegrove et al., 2006; Mallo et al., 2010; Sánchez-Herrero, 2013; Papagiannouli and Lohmann, 2015; Hrycaj and Wellik, 2016; Domsch et al., 2020). They are present in cnidarians with ancestral elements of the anterior and posterior Hox genes (Chourrout et al., 2006; Ikuta, 2011; Gaunt, 2018; Rentzsch and Holstein, 2018; Nong et al., 2020). During evolution, bilaterians acquired another set of central Hox genes and formed a complete set of genes responsible for the animal development across the anterior-posterior body axis (Chourrout et al., 2006; Hrycaj and Wellik, 2016). Usually, Hox genes are present in a cluster and exhibit spatial collinearity; the genes present in one end of the cluster are expressed in the anterior-most regions (or segments) of the developing embryo. At the same time, the genes present on the opposite end are responsible for posterior development (Gaunt, 2015). However, this is not universally true.

**FIGURE 1:**
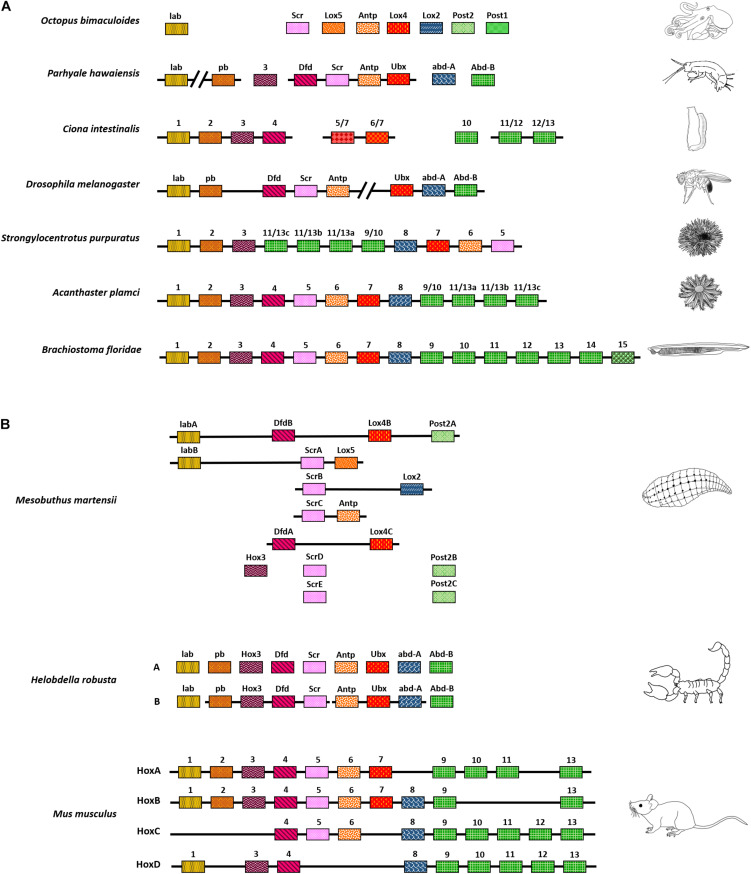
Chromosomal arrangement of Hox genes. **(A)** Variety of *Hox* arrangement observed in different animals, from a completely scattered arrangement in octopus to a fully intact *Hox* cluster in Amphioxus. **(B)** *Hox* have also undergone duplication in several organisms. They have fragmented arrangements in animals such as the freshwater leech or are present in an intact cluster, as seen in vertebrates. The depiction here is independent of phylogeny and represents the order of clustering. Also, the organisms shown here do not represent their respective phyla.

The Hox genes of California two-eyed octopus, *Octopus bimaculoides*, are completely dispersed across the genome (Albertin et al., 2015). Other than the octopus, most other bilaterians show clustering of at least two Hox genes in *cis-*. For example, in *Parhyale hawaiensis*, a crustacean, some of the *Hox* are arranged in clusters of two and four genes. However, the detailed arrangement of all Hox genes in *Parhyale* remains elusive due to the absence of long contigs (Serano et al., 2016). Even in a marine chordate, *Ciona intestinalis*, *Hox* appear to be present in an exceptionally dispersed cluster, or they could even be disseminated across the genome (Spagnuolo et al., 2003; Ikuta et al., 2004). *D. melanogaster* has a partially contiguous arrangement of *Hox*. As mentioned earlier, the Hox genes cluster of *Drosophila* is split into two complexes with 5 and 3 *Hox* in them. Both complexes are around 300 Kb in length and are separated by a distance of ∼9 Mb (Dessain and McGinnis, 1993; Rogers, 2020). Other than *Drosophila*, the red fluor beetle, *Tribolium castaneum*, has been a subject of extensive studies for patterning and evolution in insects. Both the insects have similar expressions of *Hox* orthologs in anterior-posterior segments. However, their arrangement is quite different in the genome. *T. castaneum Hox* are organized in a single tight cluster as opposed to the split found in *Drosophila* (Beeman, 1987; Telford, 2000; Brown et al., 2002; Shippy et al., 2008). In other organisms such as the starfish, *Acanthaster planci*, and sea urchin *Strongylocentrotus purpuratus*, *Hox* are present in a cluster. Still, either their orientation is altered, or they have re-ordered arrangement when compared to the majority of bilaterians that follow collinearity. In *S. purpuratus*, posterior *Hox* (*Hox11/13*) have relocated to positions analogous to central *Hox* and vice-versa (Howard-Ashby et al., 2006; Baughman et al., 2014). In contrast, the Hox genes of cephalochordate amphioxus, *Branchiostoma floridae*, are present as a single intact cluster in the order of their evolutionary homologs, along the anterior-posterior body axis. It is the most cohesive cluster of *Hox* discovered in the animal kingdom from *Hox1* to *Hox14*. Later, analysis of the region between *Hox14* and *EvxA – EvxB* led to the finding of another paralogous posterior Hox gene called *Hox15*. Thus, the cluster of Hox genes in amphioxus is by far the largest intact cluster in terms of the number of Hox genes and spans 470 Kb (Holland et al., 2008). The above examples suggest that animals have varied arrangements of Hox genes as they underwent multiple combinations of convergent and divergent evolutionary processes throughout the tree of life (Figure 1A).

In several organisms, Hox genes are present in multiple copies of paralogous genes. For instance, the cluster of Hox genes in the annelid *Helobdella robusta* appear fragmented along with varying copies of different homeotic genes. This is especially true for anterior and central *Hox* orthologs such as *Deformed* (*Dfd*) and *Sex combs reduced* (*Scr*) present in two and five copies, respectively. The *Leech homeobox gene* (*Lox4*) is also present in two copies, whereas orthologs like *Proboscipedia* (*Pb*), *Ultrabithorax* (*Ubx*), and *abdominal-A* (*abd-A*) appear completely absent. The posterior Hox gene, *Post2*, is also present in three copies (Kourakis and Martindale, 2001).

Similarly, the Chinese scorpion, *Mesobuthus martensii*, has two sets of Hox genes, with one of the sets being more clustered than the other. Interestingly, the duplication of Hox genes in scorpions is associated with variation in and extension of the posterior-most segments of the animal, including telson (Di et al., 2015). Vertebrates have at least four different paralogous complexes of Hox genes clusters. Each complex has a different number of Hox homologs arranged in a tight cluster of ∼100 Kb. The clustering also follows spatial collinearity like its invertebrate counterparts. In addition to that, vertebrate Hox genes are also expressed in a temporally collinear manner. The genes present in one end of the complex are expressed earlier during embryonic development and vice-versa. The different complexes work independently, as well as in concert, to fine-tune the growth of a developing embryo (Figure 1B; Burke et al., 1995; Medina-Martínez et al., 2000; Suemori and Noguchi, 2000; Spitz et al., 2001; Kmita et al., 2005; Tschopp et al., 2009; Yamada et al., 2021).

In conclusion, the Hox genes are present in different positions and numbers across the genome, from an atomized and dispersed manner in octopus to cleanly clustered complexes in vertebrates (Figures 1A,B). It, therefore, becomes important to understand the significance of clustering and ordering of Hox genes in different organisms. Many of the available genome sequences still lack chromosome level assemblies. With the advancement of long-read nanopore sequencing and the use of proximity ligation assays like Hi-C, it is possible to achieve chromosome level assemblies (Wang et al., 2014; Kadota et al., 2020). The ongoing earth biogenome project shall benefit from these techniques, and analysis of Hox genes arrangement in different animals will help us better understand their clustering and ordering throughout the tree of life (Lewin et al., 2018). An in-depth overview of known Hox genes clusters and their arrangement across different organisms is nicely covered in a review by Stephen Gaunt (2018).

## *Drosophila Hox* Complex: A Split That Unified the Field

**FIGURE 2:**
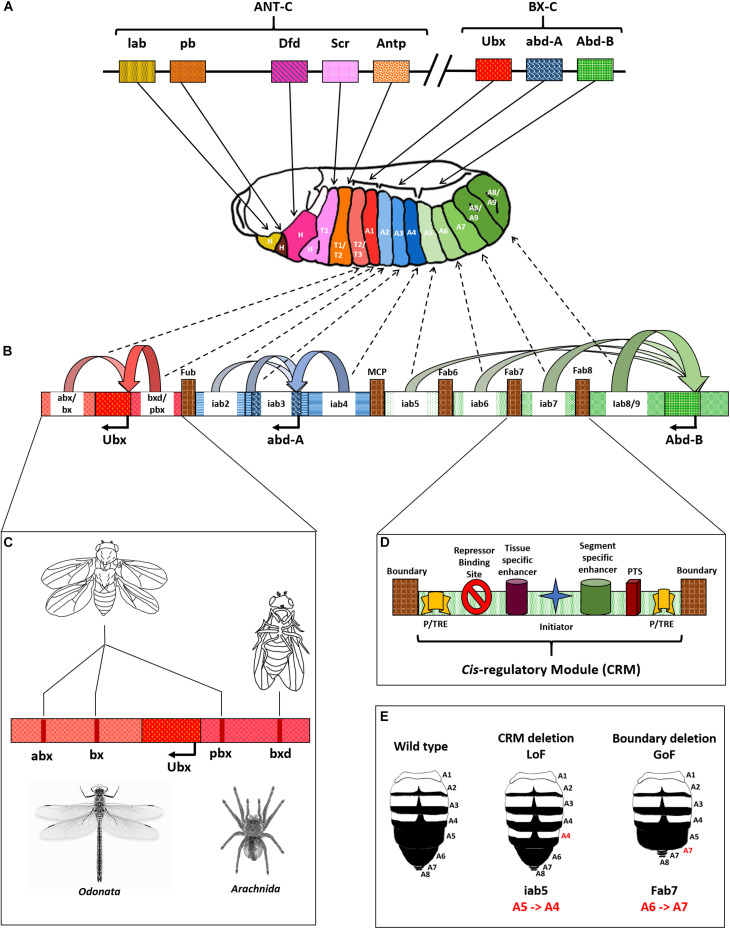
*Drosophila* Hox complex and regulation of BX-C. **(A)** *Drosophila* Hox genes are split into two complexes, Antennapedia complex (ANT-C), and bithorax complex (BX-C), as shown. Each gene is responsible for providing identity to a specific segment, as indicated by bold arrows. **(B)** *Cis*-regulatory modules (CRMs) of the BX-C cause differential expression of Hox genes in a segment-specific manner. The genes influenced by their CRMs are shown as curved arrows with respective colors, and the dotted arrows indicate the segments they influence. **(C)** Representation of *cis*-regulatory module with different elements including boundary/insulator, Polycomb/Trithorax Response Elements (P/TRE), and promoter tethering sequences (PTS). **(D)** Deletion mutations in CRMs of *Ubx* leading to phenotypes that look similar to an odonate, like dragonfly or an arachnid, like spider. **(E)** Loss and gain of function mutations in the abdominal region of *Drosophila* due to deletions of CRMs or BEs.

Homeotic genes were discovered by Ed Lewis in *D. melanogaster* in the latter half of the 20th century (Lewis, 1978). There are two clusters of these genes in the fruit fly, the Antennapedia complex (ANT-C) and the bithorax complex (BX-C). The ANT-C is responsible for the identity of anterior segments of the fly from the head through thoracic segment 2 (T2) and has five Hox genes. In the proximo-distal arrangement concerning centromere-telomere, these genes are ordered as *labial* (*lab*), *Proboscipedia* (*Pb*), *Deformed* (*Dfd*), *Sex comb reduced* (*Scr*), and *Antennapedia* (*Antp*). The BX-C has three genes in the centromeric proximo-distal order of *Ultrabithorax* (*Ubx*), *abdominal-A* (*abd-A*), and *Abdominal-B* (*Abd-B*). These genes provide identity to the posterior two-thirds of the fly’s body axis from T3 to abdominal segment 8/9 (A8/9), which is the terminal segment in the fly (Figure 2A; Lewis, 1978, 1998; Kaufman et al., 1990; Dessain and McGinnis, 1993). It is noteworthy that there are various CRMs for each Hox gene in the fly. These CRMs consist of numerous regulatory elements, including enhancers, initiators, insulators or boundary elements (BE), Polycomb/Trithorax response elements (P/TRE), and promoter tethering sequences (PTS), that together orchestrate the segment-specific expression of these genes (Figures 2B,D; Celniker et al., 1989, 1990; Simon et al., 1990; Sánchez-Herrero, 1991; Castelli-gair et al., 1992; Muller and Bienz, 1992; Mishra and Karch, 1999; Bender and Hudson, 2000; Calhoun and Levine, 2003; Lin et al., 2003; Mihaly et al., 2006; Iampietro et al., 2010; Li et al., 2015).

There are nine CRMs of the three BX-C genes in the order *anterobithorax/bithorax* (*abx/bx*) and *bithoraxoid/postbithorax* (*bxd/pbx*) for *Ubx*, *infra-abdominal2* (*iab2*), *iab3* and *iab4* for *abd-A*, and *iab5*, *iab6*, *iab7*, and *iab8/9* for *Abd-B*. Each of the CRM drives segment-specific expression of the associated gene in embryonic parasegment 5 (PS5) through PS14, corresponding to segments T3 through A8/9 in the adult fly. Deletions of CRMs cause loss of function (LoF) phenotypes for the associated Hox genes and lead to anteriorization of respective segments. For example, deletion of *iab5* causes homeotic transformation of A5 to A4. The mutant has two copies of A4 after A3 that follow the normal occurrence of A6, A7, and genitalia (A8/9) (Figure 2E; Martinez-Arias and Lawrence, 1985; Peifer and Bender, 1986; Turner and Kaufman, 1987; Galloni et al., 1993; Casares and Sanchez-Herrero, 1995; Hendrickson and Sakonju, 1995; Martin et al., 1995; Bender and Hudson, 2000; Bae et al., 2002; Estrada et al., 2002; Deutsch, 2004; Mihaly et al., 2006; Starr et al., 2011). Further, chromatin domain boundaries separate the CRMs of the BX-C. These include Front-ultraabdominal (Fub) that separates *bxd/pbx* from *iab2*, Mis-cadastral pigmentation (MCP) separating *iab4* and *iab5*, Frontabdominal6 (Fab6) between *iab5* and *iab6*, (Fab7) demarcating the domains of *iab6* and *iab7*, followed by (Fab8), which is present between *iab7* and *iab8/9* (Figure 2B). These BEs maintain the autonomous domains of functioning for different CRMs and genes. In contrast to the LoF phenotypes of CRM deletions, the deletions of BEs cause gain of function phenotypes for the associated Hox genes leading to posteriorization of the related segments. This phenotype is due to the ectopic activation of posterior CRM and its prevalence over the anterior one. For instance, deletion of the chromatin domain boundary, Fab7 leads to the homeotic transformation of A6 to A7 as depicted in Figure 2E (Simon et al., 1990; Karch et al., 1994; Hagstrom et al., 1996; Zhou et al., 1996; Mihaly et al., 1998; Mishra and Karch, 1999; Muller et al., 1999; Barges et al., 2000; Schweinsberg et al., 2004; Bender and Lucas, 2013; Postika et al., 2018, 2021). Furthermore, multiple P/TREs adjacent to the BEs and inside CRMs maintain the repressed or activated states of associated CRMs. A combination of boundaries and PREs maintain the distinct autonomy of CRMs wherein the PREs are known to function via DNA kissing (Simon et al., 1993; Chan et al., 1994; Mishra et al., 2001; Vazquez et al., 2006; Lanzuolo et al., 2007; Bantignies et al., 2011; Négre et al., 2011; Singh and Mishra, 2015). The CRMs of BX-C are also present in a spatially collinear manner in tune with their associated genes (Lewis, 1978, 1998; Maeda, 2006). Figure 2 summarizes the arrangement of *D. melanogaster Hox* and the elements of the bithorax complex. Notably, the significance of positioning of CRMs in a collinear manner is still elusive. A significant merit could be the sequential regulation of the Hox genes by upstream regulators as proposed in the open for business model of BX-C regulation (see next section) (Maeda and Karch, 2007, 2015; Kyrchanova et al., 2015).

Since Hox genes provide identities to a developing segment, altering the levels of these genes can tip the scale in favor of distinct traits gained or lost during evolution, albeit they are not the sole drivers of the process (Ho et al., 2009; Holland, 2015). For example, mutations in the CRMs of the *D.mel Ubx* gene manifest fascinating phenotypes. A triple deletion mutant for *abx*, *bx*, and *pbx* causes homeotic transformation of T3 into a copy of T2. The T3 of flies possesses a pair of rudimentary wings called halteres that help maintain balance during flight (Lewis, 1978; Dickinson et al., 1999; Yarger and Fox, 2016). In the triple mutant, the halteres get completely transformed into wings, and the posterior thorax attains the morphology of the anterior one resulting in a fly with four wings, compared to a pair of wings and halteres in normal conditions (Figure 2C). Since the CRMs maintain required levels of *Ubx* in T3, their absence leads to a lack of expression of the gene. This loss of function causes T3 to anteriorize into a copy of T2 (Little et al., 1990; Martínez-Laborda et al., 1996). It was a remarkable achievement for two reasons – (1) All three mutations were within a span of 100 kb of each other and were therefore extremely difficult to obtain in *cis-* through the genetic crossing. (2) The fruit fly, a dipteran, looks strikingly similar to an odonate like dragonfly or damselfly with four distinct wings (Lewis, 1978). A combination of three inter-genic mutations led to the development of body morphology that diverged almost 500 million years before the arrival of dipterans (Figure 2C; Misof et al., 2014). Similarly, flies hemizygous for *bxd* have a partial transformation of A1 into a copy of T3, resulting in a fly with four pairs of legs instead of three. This feature is similar to an arachnid that includes spiders, scorpions, and ticks (Figure 2C; Shultz, 1989).

**FIGURE 3:**
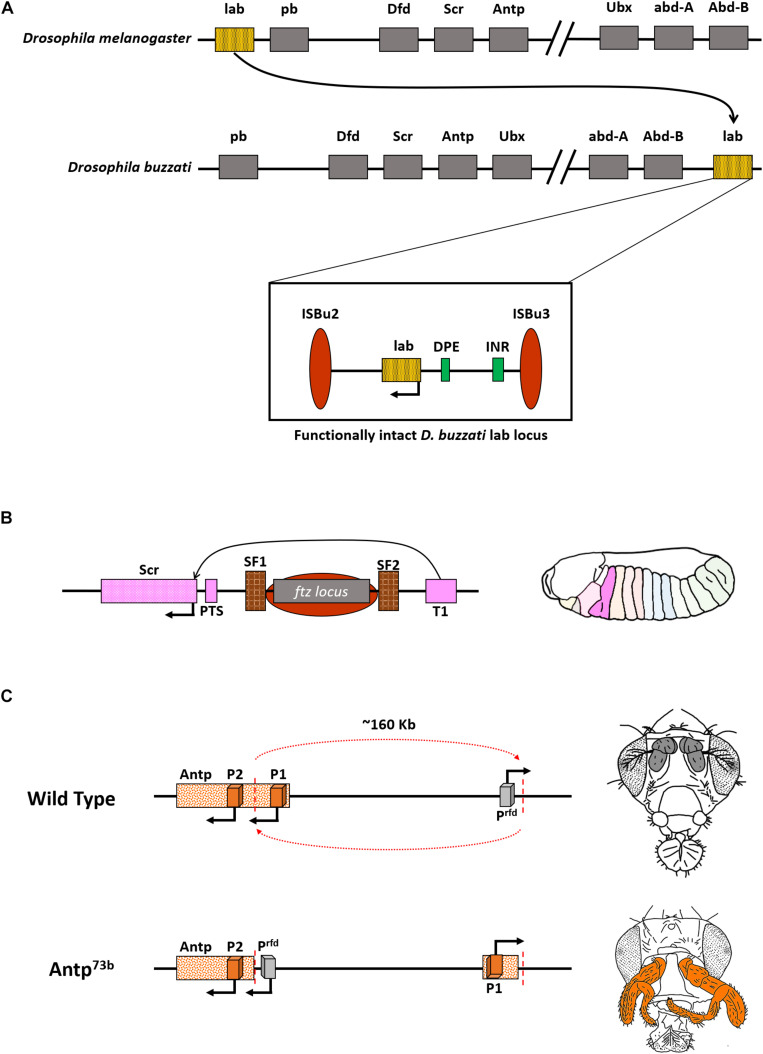
Functional CRMs can accommodate changes in gene order. **(A)** Representation of Hox in *D. melanogaster* and *D. buzzati*. Note that the gene *labial* (*lab*) is present next to *Abdominal-B* (*Abd-B*), and the split is between *Ubx* and *abd-A*, wherein *Ubx* is now a part of ANT-C. **Inset:** Functional intactness of *lab* locus despite rearrangement owing to two transposons, ISBu2/3, that flank the regulatory cassette of the gene. **(B)** Illustration depicting regulation of Scr gene bypassing ftz locus. The ftz gene and regulators are flanked by boundary elements SF1 and SF2. T1 is the enhancer for Scr. **Right:** Domain expression of Scr in a normal condition. **(C)** Inversion involving *Antp* (*Antp^73*b*^*) spanning ∼160 Kb juxtaposes the gene to an ectopic promoter that drives its expression in antennae and arista of the flies (colored bold gray in wild type fly). This leads to the homeotic transformation of antennae to legs (highlighted in orange).

The presence of intact CRMs juxtaposed with genes would ensure that they provide coordinated expression during embryonic development. This is evident from the case of *Drosophila buzzati*, where the gene *labial* (*lab*), an anterior gene, is relocated to a position nearer to *abd-A* and *Abd-B*, the genes that define the posterior development of the fly. Nevertheless, the expression pattern for all Hox genes remains similar to *D. melanogaster*. The rearrangement of the *lab* locus was attributed to the presence of two transposable elements, ISBu2 and ISBu3, that stabilized over generations. These transposons together flank the gene *lab* and its associated non-coding elements. So, the overall arrangement of transposons, associated non-coding elements, and absence of any other coding gene indicate the functional intactness of the locus (Negre et al., 2003). The *D. buzzati lab*, hence, still expresses in the anterior part of the body, unlike its neighbors *abd-A* and *Abd-B* (Figure 3A).

The process of *cis*-regulation can be effectively carried out even in the presence of a non-related DNA element in between. For example, few of the *cis*-regulators of the *Dmel Scr* gene are present after a non-homeotic gene, *ftz* (Gindhart et al., 1995; Calhoun and Levine, 2003). The *ftz* gene is, however, flanked by two strong boundary elements SF1 and SF2, that presumably loop out the gene and its associated regulators, thus, facilitating proper interaction of *Scr* enhancer, T1, with the *Scr* gene (Figure 3B; Nègre et al., 2010; Li et al., 2015). This process is similar to insulator bypass events, observed in BX-C in the presence of boundary elements like MCP, Fab-7, or Fab-8 (Sipos et al., 1998; Mishra and Karch, 1999; Kyrchanova et al., 2011, 2015, 2019).

Though the genes and CRMs can together relocate to various positions across the genome or be reorganized by chromatin domain boundaries, an arbitrary split in the middle of CRMs is deleterious. This is apparent from the famous *Antennapedia* mutant, *Antp^73*b*^*. The *Dmel Antp* gene has two promoters, P1 and P2. A breakpoint of 45 Kb upstream of P2 separates it from P1 and results in an inversion that repositioned P2 around 160 Kb away from its original locus. The inversion also leads to repositioning a non-specific promoter of an uncharacterized gene, *responsible for dominant* phenotype (*rfd*), in the *Antp* locus. This promoter (P^rfd^) causes ectopic expression of *Antp*, leading to a gain of function phenotype, characterized by the homeotic transformation of antennae and arista in the fly into a pair of legs (Figure 3C). Embryos homozygous for *Antp^73*b*^* die early during development. These findings support the theory that ectopic promoters can drive the expression of nearby genes in a non-specific manner (Laughon et al., 1986; Schneuwly et al., 1987). Along with gaining insights into the regulation of BX-C, *Scr* locus, and *Antp* associated dominant phenotype, the understanding of the Hox complex in *Drosophila* was pivotal for dissecting the embryonic development of an organism and also led to a better understanding of crucial facets of gene regulation (Figure 3). A plethora of subsequent studies in the following decades after the discovery of *Hox* revealed their existence in all bilaterians as well as cnidarians (Burke et al., 1995; Brooke et al., 1998; Peterson et al., 2000; Ferrier and Holland, 2001; Samadi and Steiner, 2010; Ikuta, 2011; Gaunt, 2018). Deciphering the functioning of the *Drosophila* Hox genes complex, in particular, the BX-C, led to a better understanding of embryonic development, molecular biology, patterning, and evolution. Welcome Bender rightly proposed that the regulation of BX-C should enter textbooks at par with *lac* operon, phage transcription, and yeast mating-type (Bender, 2020).

## Clustering, *Cis*-Regulation, and Remote Controls of *Hox* Expression

Segment-specific activation and expression of Hox genes are important for segment identity. Transcription factor coding genes including Gap, Pair-rule, and segment polarity genes act upstream of Hox genes and regulate their expression via associated CRMs in insects (Capdevila and Garcia-bellido, 1981; Reinitz and Levine, 1990; Kornberg and Tabata, 1993; Casares and Sanchez-Herrero, 1995; Drewell et al., 2014). As mentioned earlier, there are nine CRMs in the BX-C that direct expression levels and patterns of *Ubx*, *abd-A*, and *Abd-B* in a segment-specific manner.

**FIGURE 4:**
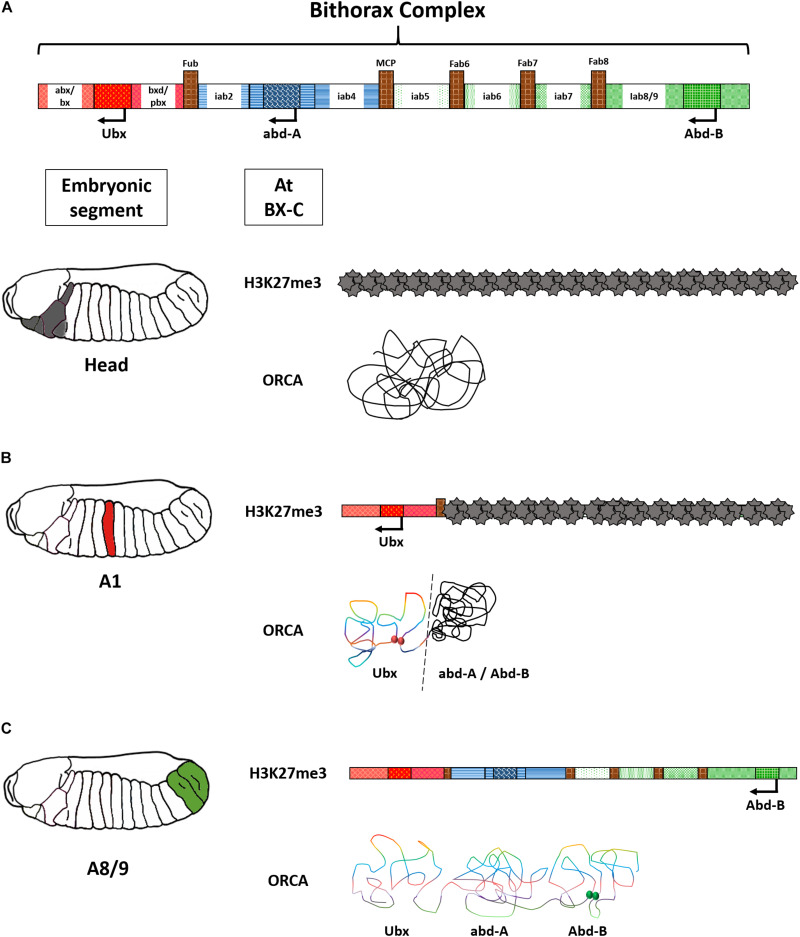
Open for business model of the bithorax complex. **(A)** H3K27me3 marks on the bithorax complex in the head reveal complete repression of the locus. The same is corroborated by a representation of the ORCA image that shows clustering of the entire BX-C in one domain. **(B)** In A1, only the *Ubx* domain is derepressed and forms a distinct loop. Red spheres indicate contact points of Ubx enhancers with its promoter. **(C)** In A8/9, the entire BX-C is de-repressed and forms multiple loop domains. Green spheres indicate contact points of Abd-B enhancers with its regulators. The above image is a conceptual representation of data published by Bowman et al. (2014) and Mateo et al. (2019).

These regions are tightly regulated. Probing the chromatin landscapes of Hox locus has shed some light on their mode of regulation. Segment-specific ChIP-seq for H3K27me3 repressive marks on *Drosophila* BX-C has pinpointed regions that were sequentially de-methylated from anterior to posterior segment in the fly embryo. For instance, in the head, the BX-C is marked with H3K27me3, coinciding with the absence of expression of all the three genes in the complex. While in A1, the *Ubx* domain lacked H3K27me3 marks corroborating with the expression status of *Ubx* in the segment. However, the other two genes of the same complex, *abd-A*, and *Abd-B*, were still carrying the repressive marks (Figures 4A–C). This indicated a segment-specific “opening” of BX-C CRMs as one would move from the anterior to the posterior regions in the fly axis and was aptly called the open for business model of the bithorax complex (Bowman et al., 2014; Maeda and Karch, 2015). This model was later reinforced by visualization of chromatin landscape of the BX-C using the optical reconstruction of chromatin architecture (ORCA) technique. It deploys sequential probing of the region of interest on a chromosome, which in this case was ∼320 Kb of the BX-C, by fluorescent probes. The probes are hybridized and washed in a series. They are then coupled with continuous imaging using two customized microscope platforms optimized for HiLo illumination (Mateo et al., 2019). ORCA is advantageous over conventional confocal microscopy due to the single-molecule resolution possible using the said platform. Like conventional imaging, samples are uniformly illuminated, but a high pass filter rejects the illuminated regions that are outside focus. The extracted data is fused with low-frequency in-focus illumination to render a spatially resolved, sharp image (Ford et al., 2012). ORCA of BX-C revealed interactions of segment-specific enhancers with the associated promoters in an *in vivo* context. Regions devoid of repressive marks were forming a distinct loop, while the ones that remained repressed were forming another closed loop domain (Figures 4A–D; Mateo et al., 2019). The clustering of the CRMs and genes in a relatively short region can be an efficient way to moderate Hox levels. In the unusual cases of Hox arrangement like octopus, sea star, or sea urchin, deciphering 3D genome architecture would provide crucial insights into their functioning.

The range of available model organisms limits our current knowledge. Nevertheless, *in silico* and synthetic biology approaches can help in designing experiments of physiological and evolutionary relevance. Crocker et al. (2017) modeled functional enhancers based on the binding sites of various transcription factors across different species of *Drosophila*. They produced several synthetic enhancers which could be validated *in vivo* in a developing fly embryo. However, only a limited number of the predicted enhancers could emulate the expression ability of the native ones (Crocker et al., 2017). This could be because of additional factors like insulators and Polycomb/Trithorax response elements (P/TREs) that contribute to regulatory aspects of the genome. Toward this, Srinivasan et al. developed *in silico* tools to predict chromatin domain boundaries and P/TREs in *Drosophila* and other insects (Srinivasan and Mishra, 2012, 2020). With the ever-expanding availability of genome sequences, such tools can be extended to model regulation of genes, including *Hox*, in a diverse set of organisms (Lewin et al., 2018).

Notably, many of the regulatory elements of the genome, like enhancers and insulators, are known to interact with regions that are several Mbs apart (Long et al., 2016). Despite that, the clustering of CRMs and Hox genes in complex organisms suggests a very strong functional consequence. It is speculated that the order of genes within the Hox complex is important for proper body axis development. However, it may be the order of CRMs that might be equally important.

**FIGURE 5:**
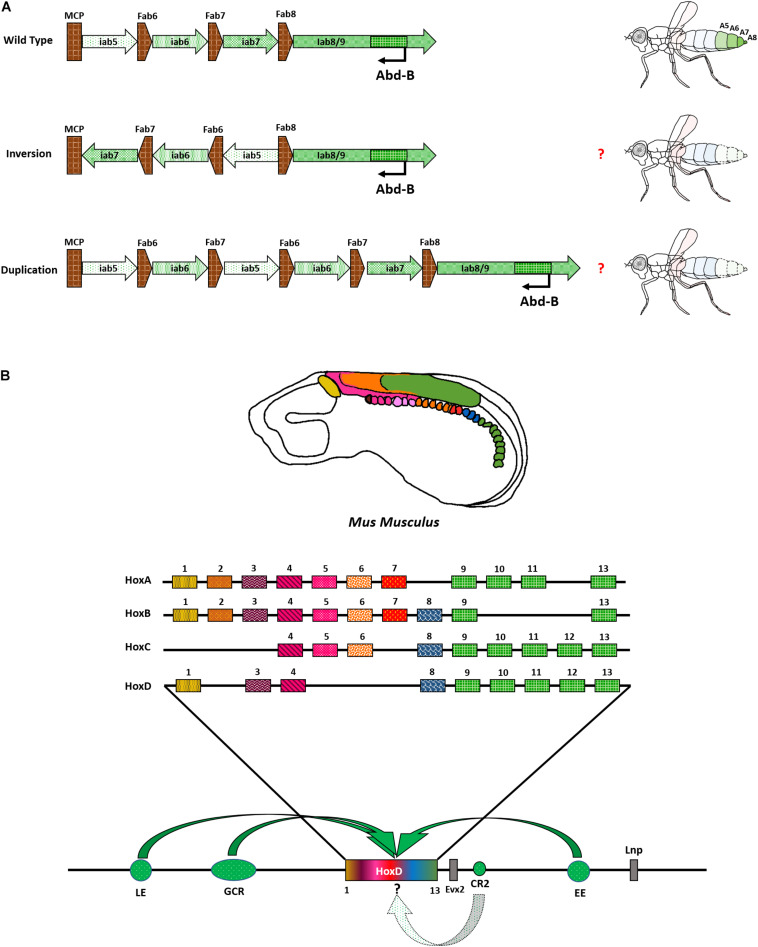
Re-arrangement of *Abd-B* locus and vertebrate Hox complex with CRMs. **(A)** Representation of proposed experiment to re-arrange the CRMs of Abd-B locus in the BX-C. **(B)** Hox genes are expressed as indicated in *Mus musculus.* HoxD locus is shown as a representation of regulatory modules associated with Hox complexes. Bold, curved arrows indicate their approximate presence and interaction with Hox complex (not to scale and point pre*cis*ely on a particular Hox). The role of CR2 in modulating Hox is still unknown and is represented by a dotted arrow.

An intriguing region to understand the significance of relative positioning of CRMs can be the *Abd-B* locus in the BX-C. Each of the *iab*s (CRM) in the region is demarcated by chromatin domain boundaries (BEs) (Figures 2B,D,E). For example, *iab5* specifies PS10 (A5) identity and is followed by a BE, Fab6. The BE separates *iab5* from the next CRM *iab6*, which specifies PS11 (A6) of the fly embryo, thus ensuring autonomous domains of the two CRMs (Galloni et al., 1993; Lewis, 2007; Bender et al., 2011; Postika et al., 2021). Together, the four *iab*s of the *Abd-B* locus provide identities to four abdominal segments in the fly from A5 to A8 (terminalia). Hence, the number of these CRMs and their relative positioning in the genome is collinear with the segment they provide identity (Maeda, 2006; Lewis, 2007; Kyrchanova et al., 2015; Maeda and Karch, 2015). Furthermore, many of the BEs are known to function in an orientation-dependent manner. However, most of these studies are done in a transgenic context or a narrow region within the BX-C (Galloni et al., 1993; Martin et al., 1995; Bender and Hudson, 2000; Kyrchanova et al., 2016, 2019). In principle, the iterative arrangement of CRMs and BEs in the *Abd-B* locus is a compelling case to decipher their role in complex systems. An interesting experiment would be to generate targeted inversions and duplications of CRMs in the BX-C and examine the resulting novel phenotypes. The re-arrangements should be developed in a manner that does not affect binding sites for transcription factors, repressors, or chromatin remodelers obtained from existing ChIP data in the modENCODE consortium (Celniker et al., 2009; Nègre et al., 2010; Négre et al., 2011). Moreover, these re-arrangements should not fuse the domains of two nearby genes or known regulators, as indicated in Figure 5A. One could harness the potential of Cre-LoxP or FLP-FRT systems to bring about these changes. The recombinase recognition sequences can be knocked in at specific sites using CRISPR/Cas9 (Li et al., 2020). For instance, a reorganized locus with MCP followed by *iab7*, *iab6*, and *iab5* will offer a new playground for *cis*-/*trans*- factors to regulate *Abd-B*. The rearrangement would render *iab7* flanked by MCP and Fab7 in opposite directions, whereas Fab6 and Fab8 boundaries will flank *iab5*. Although the relative positioning of *iab6* would remain the same, but, according to the open for business model of BX-C regulation, either *iab5* and *iab8* will become accessible to *Abd-B* promoter, or *iab7* will be accessible irrespective of re-ordering in PS11 (prospective A6). Such an experiment can unfold the aspects of directionality, ordering, and relative positioning of CRMs within the particular Hox gene locus. Similarly, generating duplications of CRMs like *iab5* and *iab6* will provide a better understanding of the significance of the number of CRMs required to specify a segment (Figure 5A). Inversions in several *cis*-regulators in vertebrates have revealed the significance of positioning distal enhancers concerning Hox genes (Kmita et al., 2000; Zakany et al., 2004). Site-specific rearrangements and deletions of vertebrate *cis*-regulators revealed modularity associated with their arrangements and caused changes in the topologically associated domains (TADs) in which they reside. This leads to the ectopic expression of Hox genes in non-specific regions of the limb, thereby suggesting a significant role of the positions of CREs (Fabre et al., 2017). Since BX-C has a spatially collinear arrangement of the CRMs with a clear understanding of their components, the re-engineered locus will provide a deeper understanding of the evolution of CRM positioning and copy number variations (CNVs).

Overall, the situation is perhaps a bit more complex in vertebrates. They have a minimum of 4 *Hox* complexes distributed across different chromosomes. Each complex has its own set of regulators. Their embryonic expression follows spatio-temporal collinearity. This means that the genes present toward one end of the cluster are expressed earlier in the anterior regions. The genes present toward the other end of the cluster are expressed later in time in the posterior regions. So, there is an added temporal aspect of regulation in addition to the pre-existing spatial one. Furthermore, the clustering of *Hox* is more pronounced in vertebrates, with no non-homeotic genes present in the complex. The intergenic distance is also drastically reduced and the entire *Hox* complex resides within a span of ∼100 Kb. In contrast, both *Hox* complexes in *Drosophila* are larger than 300 Kb. The reduction in the size of the complex can be attributed to the positioning of CRMs of vertebrate *Hox*, outside the cluster on either end, several Kbs away. These regions constitute the global control regions (GCRs), early enhancers (EE), late enhancers (LE), and many other uncharacterized regulatory elements (Soshnikova, 2014). The tight clustering of *Hox* in vertebrates might also help in robust regulation during secondary axis formation in the limbs when the collinearity is replayed (Soshnikova and Duboule, 2009; Mallo et al., 2010; Mallo and Alonso, 2013; Soshnikova, 2014). Some studies have shown several regions that are ultra-conserved near the *HoxD/Evx* locus of vertebrates. One of these regions, called conserved region 2 (CR2), was shown to have an early enhancer but late repressor activity in a transgenic context in zebrafish, *Danio rerio* (Sabarinadh et al., 2004; Kushawah and Mishra, 2017). The exact mechanism and mode of function of these elements are still unknown. It is also not known whether these regions have an impact on Hox genes. Deletions of these regions in several combinations can help us dissect their significance (Figure 5B).

The spatio-temporal regulation of Hox genes in vertebrates has some fascinating offshoots. Marsupials like Tammar wallaby, *Macropus eugenii*, have delayed expression of posterior Hox genes, *HoxA13* and *HoxD13*, attributed to weaker hind limbs in newborn animals. The forelimbs are relatively stronger, which helps them to climb the brood pouch of their parent. The delayed expression of the posterior *Hox* is yet another example of modularity and differential expression, possibly due to differences in clustering and accessibility of CRMs which can be accessed via the genome sequence available for marsupials (Chew et al., 2012; Deakin, 2012).

Similar variations of spatio-temporal regulation can be observed in simpler chordates like amphioxus. Despite being in a tight cluster, the spatially collinear expression of Hox genes is perturbed in *Branchiostoma floridae*. *Hox6*, a central Hox gene, expresses almost ubiquitously across the neural tube, posterior to the cerebral vesicle. While *Hox14*, a posterior Hox gene, is expressed in the most anterior cerebral vesicle. Furthermore, *Hox14* mRNA is also detected in the pharyngeal endoderm. Interestingly, levels of *Hox6* vary greatly in closely related species. Unlike *B. floridae Hox6*, which shows a uniform expression throughout the neural tube, the *B. lanceolatum* homolog expresses in a spatially restricted manner (Pascual-Anaya et al., 2012). This indicates subtle modulations of HOX levels in closely related species and is similar to changes observed in invertebrates. Deep sequencing of flanking regions of Hox loci in multiple organisms along with a Bag-of-Motif analysis to understand protein-DNA interactions can shed light on putative regulatory mechanisms associated with the clustering of CRMs.

In simpler organisms like annelids or mollusks, the arrangement of *Hox* thus seems to be dispensable, but with the evolution of complexity, clustering becomes a necessity for co-regulation.

## Modulating Hox in Arthropods

The property of a system to separate and re-integrate its components to form a viable system is called modularity. Subtle changes in Hox expression can quickly orchestrate the evolutionary modularity. The studies are not limited to fruit flies. In an amphipod crustacean, *Parhyale hawaiensis*, the interplay between various Hox genes and their ability to act independently was comprehended by a series of sophisticated experiments involving manipulation of Hox levels (Liubicich et al., 2009; Martin et al., 2016; Sun and Patel, 2019).

**FIGURE 6:**
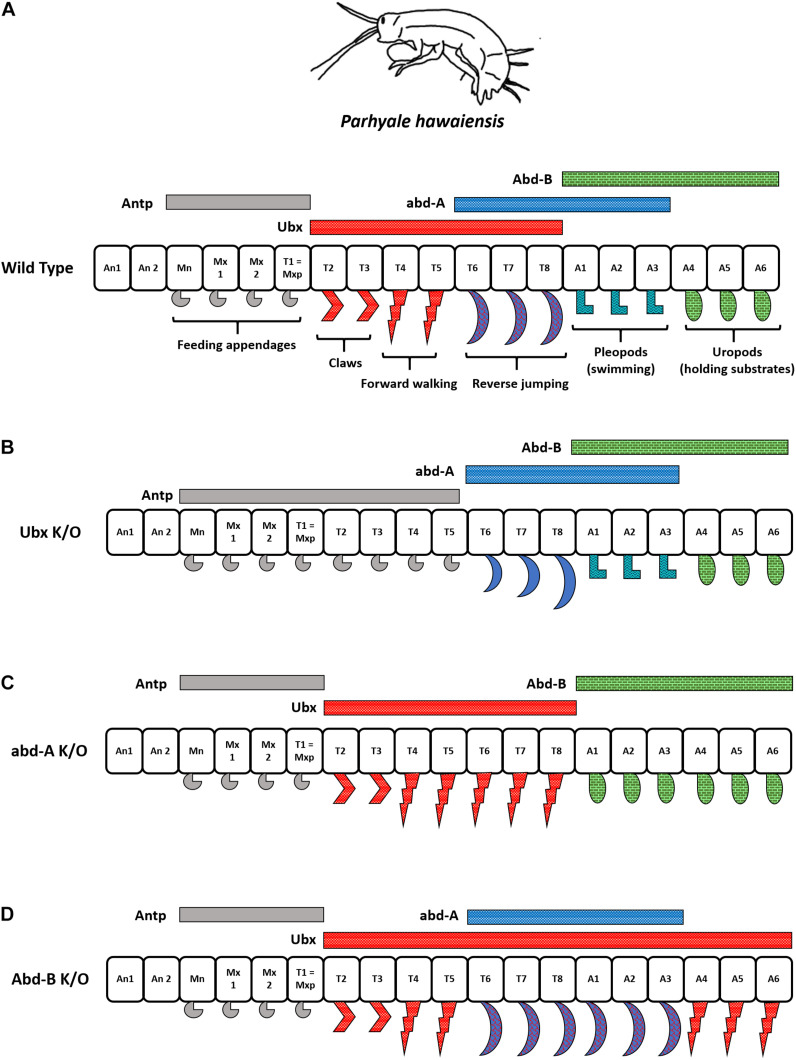
Interplay of different Hox genes in *Parahyale hawaiensis*. **(A)** Hox expression in *Parhyale hawaiensis*. Note that *abd-A* has overlapping regions of functioning with *Ubx* as well as *Abd-B*. **(B)** *Ubx* knock-out animals show anteriorization of forward walking legs. **(C)** abd-A knock-out animals show anteriorization of reverse walking legs and posteriorization of swimming appendages. **(D)** Abd-B knock-out animals show anteriorization of swimming appendages into reverse walking legs and uropods in forward walking legs.

The amphipod is bilaterally symmetrical and has multiple segments with specialized appendages. A group of metameric segments evolved to perform a common function is called tagma, and the associated evolutionary process is called tagmatization (Abzhanov and Kaufman, 2000). The arrangement of appendages in the order of their occurrence from anterior to posterior segments in *Parhyale* is as follows – feeding appendages (mandible, Mn and maxillipeds Mx, or, gnathopods), claws (T2–T3), forward (T4–T5), and reverse (T6–T8) walking legs (pereopods), swimming appendages (pleopods or swimmerets) in the segments A1 to A3, and appendages for holding substrates (uropods) formed in A4–A6. A simple representation of *P. hawaiensis* tagmatization is depicted in Figure 6A.

Recent advances in CRISPR-based gene editing have allowed researchers to perform knock-out experiments in *P. hawaiensis*. Martin et al. (2016) knocked out *Ph Ubx* by CRISPR/Cas9 and observe that the locomotor appendages acquire the identity of feeding appendages (T2–T5 → Mn/Mxp). Further, knocking out a posterior Hox gene *Ph abd-A*, which would otherwise be responsible for forming reverse walking legs in the crustacean, now has them transformed into a copy of forward walking legs (T6–T8 → T4); Figure 6B. This was as expected from previous studies in *Drosophila* that indicate the anteriorization of segments in the absence of posterior *Hox*; a property called the posterior prevalence of Hox genes. However, another class of legs called pleopods or swimming appendages was transformed into a copy of posterior appendages, uropods (A1–A3 → A4), in the Ph abd-A knock-out animals. This was an apparent effect of the additive function of *Ph abd-A* to regulate segment identity in either direction along the AP axis. In the anterior regions, *Ph abd-A* works with *Ph Ubx* to develop segments with reverse walking legs (T6–T8). In the posterior regions, *Ph abd-A* functions with *Ph Abd-B* to develop swimming appendages (A1–A3) as depicted in Figure 6C. Strikingly, *knock-out* of an even more posterior gene *Ph Abd-B* displayed a spectacular non-linear transformation of uropods into copies of forward walking legs but not swimmerets (A4–A6 → T5; not A3); Figure 6D. This suggested that the ABD-B represses *Ph Ubx* in the posterior segments of the *Parhyale* from A1 to A6, whereas *Ph abd-A* expression is independent of the ABD-B levels. *Ph Abd-B* knock-out animals had derepression of *Ph Ubx* in the posterior segments, leading to extreme transformation into forward walking legs. The segment with overlapping domains of *Ph Ubx* and *Ph abd-A* developed reverse walking legs in *Ph Abd-B* knock-outs while swimmerets were altogether absent from the organism (A1–A3 → T8); Figures 6A,D. The studies from the crustacean suggest that alongside collinear expression of *Hox*, the co-regulation, inter-regulation, and cross-talk between different HOX cause varying phenotypes. The interplay between these genes brings about diversity in the animal kingdom (Martin et al., 2016).

In addition to the AP axis, a handful of studies also show the role of Hox genes in LR and DV axis determination (Thickett and Morgan, 2002; Mohit et al., 2006; Coutelis et al., 2013). An exemplar is the *Dmel Abd-B* regulating *MyosinID* (*MyoID*), a protein responsible for complete dextral (clockwise 360°) rotation of spermiduct around hindgut during metamorphosis. *Abd-B* knocked down flies show partial sinistral (anti-clockwise) rotation to varying degrees that causes male sterility due to rotation of external genitalia (Spéder et al., 2006; Coutelis et al., 2013). Crustaceans such as *P. hawaiensis* develop symmetrically along the LR axis, and early knock-down of *Ph Ubx* in one of the sides causes asymmetrical homeotic transformation of segments, including appendage formation. This was done by injecting morpholinos for *Ph Ubx* knockdown in one of two-celled stage embryo cells. Each cell follows its fate separately across the LR axis of development. Although the system was utilized to compare wild type versus knockdown phenotypes in the same organism (Browne et al., 2005; Liubicich et al., 2009; Pavlopoulos et al., 2009), the study also implies asymmetrical differentiation of body segments upon differential expression of Hox genes. In tune with this, in the *Xenopus* embryo, *HoxC8* expresses asymmetrically along the left-right axis of development in the lateral plate mesoderm (Thickett and Morgan, 2002). One interesting organism worth probing for Hox genes regulation and determination of the LR axis is the fiddler crab. It is a natural example of left-right asymmetry in appendage formation. The female fiddler crabs have similar-sized left and right feeding appendages. In comparison, males have one of their feeding appendages extraordinarily enlarged. They use this appendage to fight competitors during mating and display handedness (Pardo et al., 2020). A detailed understanding of Hox expression in these organisms can shed light upon the formation of segments in AP and LR axes of development.

Another example of a modified and rather intriguing appendage is the scorpion’s tail, including the terminal telson. Scorpions have undergone duplications of Hox genes, which are correlated to the heteronomy of the posterior segments (Sharma et al., 2014). Arizona bark scorpion, *Centruroides sculpturatus*, has 19 Hox genes instead of 10 in its sister groups. The dual copies are expressed in varying degrees from antero-central to telson. These include *Antp*, *Ubx*, *abd-A*, and *Abd-B*. In *C. sculpturatus*, extended-expression of the two copies of *Cs Antp* and *Cs Ubx* is corroborated with enlarged telson in a distinct shape for an appendage. Notedly, the telson is formed posterior to terminalia (anus). It would be interesting to delete one or multiple copies of each of these Hox genes and observe the changes in body patterning. The tagmatization could be affected to the extent that the body form might become less elongated, as is the case with Opiliones, harvestmen, or instigated to form a telson-less scorpion (Sharma et al., 2012). The opposite spectrum of body formation is seen in Tardigrades, in which deletion of several Hox genes correlates with their compact body plan with simpler, repetitive, and less (four) number of segments (Smith et al., 2016).

Other than the levels of HOX, structural modifications in the transcription factors can help in diverse functions. Recent experiments with flies provided evidence of functional conservation of mouse Hox genes. Singh et al. (2020) replaced *labial*, the anterior-most gene in *Drosophila* Hox complex, with *Hox1* from *Mus musculus*. Interestingly, out of the three copies of *Hox1* in the form of *HoxA1*, *HoxB1*, and *HoxD1*, only *HoxA1* could rescue the *labial* knock-out phenotype completely. They also developed animals with chimeric HOX proteins and discovered a six-amino acid C-terminal motif in *HoxA1* essential for its interaction with PBX. The ortholog-specific interaction leads to differential occupancy of *HoxA1* across the genome. This study strongly supports the notion of evolutionary modularity in Hox complex by causing structural changes in HOX that lead to similar yet functionally divergent protein products (Singh et al., 2020).

An ordered arrangement of *Hox* could have played an important role in their sequential co-regulation along the AP axis, as indicated by our understanding of BX-C regulation. One can consider Hox genes as switches to control different electrical equipment at home. They can be present anywhere across the house and can still function, as is the case of an octopus. But clustering on a switchboard gives quick, precise, and perhaps, robust control over the spatio-temporal regulation of Hox genes. This modularity could have been one reason for arthropods to surpass mollusks as the richest bio-diverse species on our planet (Benton, 2010). Many genes are co-regulated in different organisms (Snel et al., 2004). Overall, clustering is more abundant in vertebrates than invertebrates (Elizondo et al., 2009; Ferrier, 2016). Nevertheless, in addition to clustering, the ordering is an important property of Hox complexes that need to be pondered upon. The past decade has witnessed rapid advancements in our understanding of epigenetic factors, inter-genic regulators, and chromatin organization (Narlikar and Ovcharenko, 2009; Hübner et al., 2013; Allis and Jenuwein, 2016; Hug and Vaquerizas, 2018). Understanding them in the context of gene clusters, including Hox complexes, will be riveting. The Hox genes have a tremendous potential to modulate diversity by teaming up with multiple partners and setting a stage for downstream players in various axes. Different combinations of *cis-* and *trans-* regulators together bring about manifold changes that can drive evolution.

## Hox Genes: Master Regulators Beyond Embryogenesis and Homeosis

Classically, mutations in Hox genes are associated with the homeotic transformation of one body segment into another, a process called homeosis (Lewis, 1994). These mutations transformed embryonic segments, and therefore the Hox genes were established as the regulators during embryonic development (Pradel and White, 1998). However, even during embryonic development, Hox genes can still play a non-homeotic role by specifically affecting tissue homeostasis and organogenesis (Castelli-Gair Hombría and Lovegrove, 2003).

Recent studies opened new horizons to understand the role of Hox genes in an organism. A rising number of articles suggest their role beyond homeotic functions and embryonic development (Wang et al., 2009; Estacio-Gómez and Díaz-Benjumea, 2014; Gummalla et al., 2014; Rux and Wellik, 2017). In *D. melanogaster*, prolonged expression of Hox genes beyond embryogenesis is observed in developing larva and pupa (Wang et al., 2009). The three genes of the bithorax complex, *Ubx*, *abd-A*, and *Abd-B*, have defined anterior limits of expression in *Drosophila* larvae. The larva undergoes metamorphosis during pupal stages of development, ultimately eclosing as adults. One key event during this process is autophagy of most of the larval tissues, including the fat body, salivary glands, and trachea. This is further coupled with the differentiation of adult tissues that goes on till eclosion. Interestingly, all the three genes of BX-C, *Ubx*, *abd-A*, and *Abd-B* are expressed in the larval fat body (Marchetti et al., 2003). Down regulation of *Ubx* is accompanied by developmental and starvation-induced autophagy, whereas sustained expression of the Hox gene inhibits autophagy and delays metamorphosis (Banreti et al., 2014).

Like the larval fat body, larval epithelial cells (LECs) also undergo apoptosis during metamorphosis. Further, another group of cells called histoblast nest cells (HNCs) differentiates to form adult abdominal epithelial cells during pupation. Posterior BX-C genes *abd-A* and *Abd-B* have overlapping expressions in the LECs. Loss of *abd-A* impairs the apoptotic pathway in LECs and cannot be rescued by *Abd-B* alone. Moreover, HNC proliferation is hindered by *abd-A* down regulations, and the cells fail to form a complete epithelium in *abd-A* knocked down pupae. Thus ABD-A is required for both, apoptosis of LECs as well as the proliferation of HNCs to form mature abdominal epithelium in adults. The study showed that ABD-A was present in the LECs and contributed toward development together with the posterior Hox gene product ABD-B, therefore defying the property of posterior prevalence (Singh and Mishra, 2014). The study also contributed to our understanding of Hox genes’ modular capacity in an extra-homeotic and extra-embryonic manner.

Similar reports for *Abd-B* were observed in testis development, where it remains active in pre-meiotic spermatocytes. Tissue-specific knockdown of *Abd-B* in adult testes leads to a loss of maintenance of the stem cell niche required to produce normal sperms. This is because ABD-B has direct binding sites on *src42A* and *sec63*, members of *Boss* signaling involved in testes formation and sperm differentiation. *Abd-B* also has an extended effect on the orientation of centrosomes and the division rates of germline stem cells (Papagiannouli and Lohmann, 2015).

Obtaining tissue-specific cells for further studies of Hox was a Herculean task a couple of years back, as one had to do neck-breaking dissections to get ounces of desirable material. Although now, endogenous tagging of Hox genes has solved a lot of such problems. Cell sorting of fluorescently labeled HOX expressing tissues followed by multi*-omics* experiments can help us understand the genome-wide effects of HOX in adult tissues. Domsch et al. (2019) reported an endogenously tagged line for *Ubx* with GFP at the N-terminal. They utilized this resource to establish the role of *Ubx* as a major repressor of factors involved in alternate fate development in mesodermal cells. Sorting GFP expressing cells followed by ChIP and Co-IP experiments helped in a deeper understanding of modalities of *Ubx* functioning. This revealed UBX’s ability to cause repression by constantly interacting with a member of Polycomb Repressive Complex protein Pleiohomeotic (PHO) (Domsch et al., 2019). In their recent work, Paul et al. (2021) showed that not only the presence of HOX but also their dosage determines the formation of appendages – in their case, wing appendages.

The extraembryonic roles of *Hox* are more distinct in vertebrates. As early as 2003, it was evident that Hox genes play a role in non-homeotic fashion owing to the near-complete loss of hair formation in mice deficient for *HoxC13*. Although the mouse also had patterning defects, hair growth was uniformly reduced across the body (Awgulewitsch, 2003).

Recent reports showed several *HoxC* genes in the dermal papilla and associated it with regional follicle variation. In a mutant mouse line called *Koala* mutant, a 1 Mb inversion encompassed disintegration of *HoxC4* to *HoxC13* from the main complex leading to their misexpression. CTCF ChiP-seq revealed changes in levels of CTCF binding within the *HoxC* complex and perturbation of topologically associated domains (TADs) (Millar, 2018). Similar deletion studies have identified the role of HoxA genes in mammary gland formation during specific transition developmental periods (Lewis, 2000).

Owing to their multifaceted roles during and after development, levels of Hox proteins need to be tightly regulated. Misexpression of these genes has been observed in various cancers like breast cancer, melanoma, bone cancer, blood cancer, and colorectal cancer (Shah and Sukumar, 2010). Central and posterior Hox genes, *HoxA5* and *HoxD9*, have been implicated in esophageal squamous cell carcinoma. Strikingly, they were found to localize more in the cytoplasm of the mucosa cells in esophageal cancer than in the nucleus in normal cellular conditions (Takahashi et al., 2007). Similarly, ectopic expression of *HoxC6*, *HoxC11*, *HoxD1*, and *HoxD8* are observed in different cases of neuroblastoma (Manohar et al., 1996; Zhang et al., 2007). Overexpression of posterior Hox genes, particularly HoxA9-11, HoxB13, and HoxC10, is linked to the onset and tumor progression of ovarian, cervical, and prostate cancers (Jung et al., 2004; Cheng et al., 2005; Miao et al., 2007; Zhai et al., 2007). Misexpression of *HoxA9*, *HoxA10*, and *HoxC6* was also reported in cases of Leukemia caused by translocations of *mixed-lineage leukemia Mll gene*. MLL is the vertebrate homolog of *Drosophila* Trithorax (TRX) protein and helps maintain an active state of *Hox* expression in required domains (Armstrong et al., 2002; Ono et al., 2005). Hox-associated cancer is not limited to genetic mutations. Rauch et al. (2007) showed increased methylation of *HoxA7* and *HoxA9* associated CpG islands. The study highlighted epigenetic misregulation as a putative cause for Hox-related lung tumors. Likewise, promoter methylation of *HoxA5* and downregulation of *HoxA10* are associated with progressive breast carcinoma. The disease can also be caused by overexpression of *HoxB7* and *HoxB13* in these tissues (Raman et al., 2000; Chu et al., 2004; Chen et al., 2008; Jerevall et al., 2008). Misexpression studies in *Drosophila* confirmed the causal effect and established flies as a model to study Hox-associated oncogenesis. The outcome of the study was the ability of *Dfd*, *Ubx*, and *abd-A* genes to be leukemogenic when overexpressed in fat body and lamellocytes (Ponrathnam et al., 2021).

Detailed understanding of Hox genes expression and interaction during embryogenesis, tissue formation, organogenesis, and cellular homeostasis is required to delineate their functional modalities. Due to their overarching involvement in multiple processes of body formation, patterning, and evolution, Hox genes occupy a prime position in our quest toward understanding these processes in depth.

## Concluding Remarks

A long-debated topic in the field of Hox genes was their presence in the form of clusters and the property of spatio-temporal collinearity. Some recent developments also demonstrated the functioning of Hox independent of clustering. However, coordinated functioning is better when they are clustered together, as implied by the open for business model of the bithorax complex. Alterations of CRMs throughout the Hox led to a myriad of homeotic transformations. Similar genomic alterations across evolution might have experimented with Hox modules and their expression to bring about the enormous diversity we see today. Individual notes are pleasant to hear, but it’s the symphony that conveys the melody. *Hox* come together to set up the primary and secondary axes and provide constant inputs in different tissues, therefore orchestrating the developmental design sublimely. *In vivo* experiments with the latest genome editing tools and a better understanding of non-coding DNA become important for comprehending the conductors of this symphony.

## Author Contributions

RKM and NH conceived the design of the article and edited the manuscript. NH wrote the manuscript with inputs from RKM, and conceptualized and drew illustrations upon discussion with RKM. Both authors contributed to the article and approved the submitted version.

## Conflict of Interest

The authors declare that the research was conducted in the absence of any commercial or financial relationships that could be construed as a potential conflict of interest.

## Publisher’s Note

All claims expressed in this article are solely those of the authors and do not necessarily represent those of their affiliated organizations, or those of the publisher, the editors and the reviewers. Any product that may be evaluated in this article, or claim that may be made by its manufacturer, is not guaranteed or endorsed by the publisher.
